# Paraneoplastic cerebellar degeneration as a presentation of breast cancer – a case report and review of the literature

**DOI:** 10.1186/1477-7800-5-8

**Published:** 2008-04-21

**Authors:** Alia Noorani, Zaid Sadiq, Neda Minakaran, Catherine Coleman, Val A Thomas, Kefah Mokbel

**Affiliations:** 1 Breast Surgery Unit, St George's Hospital NHS Trust, London, UK; 2 Department of Pathology, St George's Hospital NHS Trust, London, UK

## Abstract

Paraneoplastic cerebellar degeneration is part of a rare spectrum of neurological syndromes whereby gynaecological, lung or breast cancers present primarily with neurological manifestations. The presence of onconeural antibodies and PET scanning help in the challenging diagnosis of these conditions but despite the treatment of the primary cancer, the prognosis for the neurological symptoms is poor.

## Background

Paraneoplastic syndromes are non metastatic neurological manifestations associated with onconeural antibodies that arise in less than 1% of patients with an underlying malignancy. These syndromes are well defined in cases of small cell carcinoma of the lung and less well known but described in cases of breast and gynaecological cancers [1,2]. We present a case of a young woman who presented with cerebellar signs, positive anti-Yo antibodes and who was subsequently found to have breast cancer. We also present a review of the literature and in particular discuss the treatment options and prognosis of this condition.

## Case presentation

The patient was a 40-year-old female who developed dysarthria and ataxia in late 2006. These symptoms lasted for two months after which she made a complete recovery. Unfortunately she relapsed in March 2007, presenting with impaired coordination, speech difficulties and ataxia and was soon after confined to a wheelchair. Breast examination was normal, and neurological examination confirmed the presence of ataxia, dysarthria and impaired coordination.

**Figure 1:**
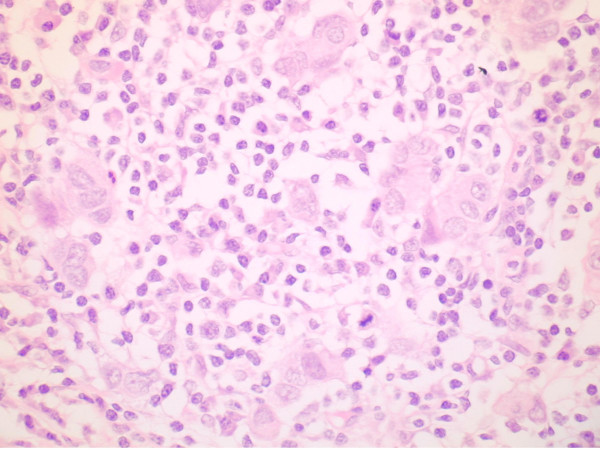
High power light microscopy showing small groups of residual tumour cells with an associated heavy lymphoplasmacytoid infiltrate.

MRI scans of the spine and brain were normal and blood tests were positive for anti-Yo antibodies. A subsequent whole body PET CT although showed no clear primary lesion, indicated an avid node in the left axilla. Bilateral mammograms were normal and ultrasound of both breasts was normal except for the pathological node in the left axilla. Fine needle aspiration of the left axillary lymph node showed metastatic carcinoma, although extensive immunocytochemical studies could not confirm or exclude breast or lung carcinoma. MRI scan of the breast showed a single 6 mm nodular lesion and a targeted ultrasound of the left breast showed several hypoechoic nodules. Core biopsies of two of the visualised nodules showed high-grade ductal carcinoma in situ and poorly differentiated invasive carcinoma (Figure 1).

The patient underwent a left skin-sparing mastectomy combined with implant-based immediate reconstruction plus axillary node clearance. Histological analysis showed a 4 cm area of grade 3 invasive ductal carcinoma and DCIS associated with extensive immunological response that had broken the tumour into small islands (Figure 1). There was no lymphovascular invasion, and 3 of 8 lymph nodes were positive for cancer. The tumour was negative for oestrogen and progesterone receptors and positive for HER2.

The patient made a good postoperative recovery and was subsequently referred for adjuvant chemotherapy and herceptin treatment and post-mastectomy radiation. Her neurological condition remained stable during treatment.

## Conclusion

Paraneoplastic cerebellar degeneration is classified as one of the paraneoplastic syndromes. These are rare, non-metastatic complications in patients with cancer, commonly gynaecological, breast or small cell lung in origin.

The pathogenesis of these syndromes is not entirely understood, but evidence suggests that certain autoantibodies expressed against tumour cells may interact with cells in the nervous system [1-3]. The anti-Yo group of antibodies belong to a group of onconeural antibodies, which are associated with breast, ovarian cancers and rarely uterine, bronchial or gastric cancers. A small proportion of patients with these malignancies go on to develop a neurological illness (<1%). Typical manifestations of this condition include ataxia, resting tremors and MRI evidence of degeneration and atrophy [4].

Anti-Yo antibodies are anti-Purkinje cell autoantibodies that act against the antigens common to the tumour and Purkinje cells in the cerebellum and are produced as an immune response to some tumours [4]. Not all patients with paraneoplastic syndromes express antibodies in their serum. Our patient expressed high titres of anti-Yo antibodies and this feature was reported in 88% of patients with paraneoplastic cerebellar degeneration [1].

Cases have been reported where cerebellar degeneration has preceded the tumour by as long as 5 years after expression of the anit-Yo antibody [5]. As in our case, FDG-PET has facilitated the early detection of cancer associated paraneoplastic syndromes [6,7].

Rojas et al evaluated the long-term outcome of PCD and anti Yo antibodies in 2000. Of a total of 34 patients with PCD and anti-Yo antibodies, tumour progression was the cause of death in 52% of cases, whilst in 29% of patients it was the neurological condition. The failure to cure the cancer in 52% of patients was due to the fact that by the time the diagnosis had been made, most tumours had already metastasised to regional lymph nodes (in our patient, 4 axillary nodes were positive for malignancy) or distant organs.

In particular, all patients with breast cancer had axillary lymph node metastases. This resembles early mediastinal lymph node metastases in patients with small cell carcinoma of the lung and anti-Hu associated syndromes. This suggests that the invasion of the regional nodes causes the tumour to invoke an immune response, which leads to the neurological syndromes. Of note, it was found in the same study, that although anti-Yo antibody associated syndromes favour the cerebellum, the disease is much more aggressive with most patients becoming bed bound within 3 months of the diagnosis [8]. The histological findings in our case indicate only partial success for the immune system fighting the primary tumour with no effect on axillary disease.

Attempts to treat the neurological symptoms on the whole, using chemotherapy, immunosuppression or immunoglobulins have not been reported to achieve any significant improvement [4]. In addition, despite successful treatment of the primary tumour depending on its stage of development, there is usually no improvement in the patients' neurological symptoms. These generally have a greater negative impact on the patients' quality of life than the underlying malignancy.

## Competing interests

The authors declare that they have no competing interests.

## Authors' contributions

KM and CC were involved with the clinical management of the patient. KM and AN were involved in the design of the study and the preparation of the manuscript. VAT carried out the histological analysis. AN, ZS and NM performed the literature search. All authors read and approved the final manuscript.

## Consent

Written informed consent was obtained from the patient for publication of this case report and any accompanying images. A copy of the written consent is available for review by the Editor-in-Chief of this journal.

## References

[B1] Rojas-Marcos I, Rosseau A, Keime-Guibert F, Ramon R, Cartalat-Carel S, Delattre JY, Graus F (2003). Spectrum of paraneoplastic neurologic disorders in women with breast and gynaecologic cancer. Medicine.

[B2] Kawasoe T, Yamamoto Y, Okumura Y, Iwase H (2006). A case report of paraneoplastic neurological syndrome associated with occult breast cancer. Breast cancer.

[B3] Gatti G, Simsek S, Kurne A, Zurrida S, Naninato P, Veronesi P, Frasson A, Millen E, Rososchansky J, Luini A (2003). Paraneoplastic neurological disorders in breast cancer. The Breast.

[B4] Dorn C, Knobloch C, Kupka M, Morakkabati-Spitz N, Schmolling (2003). Paraneoplastic neurological syndrome: patient with anti-Yo antibody and breast cancer: a case report. Arch Gynecol Obstet.

[B5] Mathew RM, Cohen AB, Galetta SL, Alavi A, Dalmau J Paraneoplastic cerebellar degeneration: Yo-expressing tumour revealed after a 5-year follow-up with FDG-PET. J Neurol Sci.

[B6] Rubello D, Vitaliani R, Rigoni MT, Rampin L, Giometto B, Casara D, Zonzin GC, Zavagno G, Capirci C, Shapiro B, Muzzio PC (2005). A rare case of paraneoplastic cerebellar degeneration discovered by whole body F-18 FDG PET. Clin Nuc Med.

[B7] Younes-Mhenni S, Janier MF, Cinotti L, Antoine JC, Tronc F, Cottin V, Ternamian PJ, Trouillas P, Honnorat J (2004). FDG-PET improves tumour detection in patients with paraneoplastic neurological syndromes. Brain.

[B8] Rojas I, Graus F, Keime-Guibert F, Rene R, Delattre JY, Ramon JM, Dalmau J, Posner JB (2000). Long term clinical outcome of paraneoplastic cerebellar degeneration and anti-Yo antibodies. Neurology.

